# The Impact of Chemotherapy on the Nutritional Status of Breast Cancer Patients

**DOI:** 10.7759/cureus.76549

**Published:** 2024-12-28

**Authors:** Haleema Kashif, Qaisar Raza, Sadia Nawaz, Kinza Imran, Syed Qasim Raza, Amir Iftikhar Malik, Muhammadah LNU, Alishba Jamil, Nimra Amjad, Sehrish Firyal

**Affiliations:** 1 Department of Food Science and Human Nutrition, University of Veterinary and Animal Sciences, Lahore, PAK; 2 Institute of Biochemistry and Biotechnology, University of Veterinary and Animal Sciences, Lahore, PAK; 3 Department of Clinical Medicine and Surgery, The Islamia University of Bahawalpur, Lahore, PAK; 4 Department of Food and Nutrition, University of Home Economics, Lahore, PAK

**Keywords:** breast cancer, changes in weight, chemotherapy treatment, dietary patterns, nutritional status

## Abstract

Background: Breast cancer is one of the most common cancers among Pakistani women. It is mostly diagnosed at stage 2, requiring chemotherapy in certain cases. Chemotherapy is of two types: adjuvant and neoadjuvant. It can be recommended at any stage of cancer, either in early stages to shrink the tumor or in late stages to improve quality of life. The side effects may include weight loss, fatigue, hair loss, sores in the mouth, loss of appetite, nausea, vomiting, difficulty swallowing, throat problems, diarrhea, infections, and anemia, which can deteriorate health. Some women may experience weight gain during treatment, which is a risk factor for cancer recurrence. Chemotherapy has many side effects that can directly affect dietary patterns and worsen nutritional status.

Objectives: This study aimed to evaluate the changes in dietary patterns of breast cancer patients during and after chemotherapy and assess how the side effects of chemotherapy affect their nutritional status.

Methodology: Data were collected from 200 breast cancer patients undergoing chemotherapy at Jinnah Hospital, Lahore, using a questionnaire and the Patient-Generated Subjective Global Assessment (PG-SGA) screening tool. The questionnaire included sociodemographic information, questions about disease, treatment, and dietary recall. PG-SGA was used to screen for malnutrition and assess nutritional status. Data collection occurred from September to December 2023.

Results: Most participants were female (99%), married (73.5%), and of low socioeconomic status (60.5%). The common stage of cancer was stage 3, and neoadjuvant chemotherapy was recommended for most participants. Effects on body weight varied, with some experiencing weight loss, others weight gain, and some no change. Side effects such as vomiting, nausea, diarrhea, dry mouth, loss of appetite, altered taste and smell, and anemia were commonly reported. Most participants were moderately malnourished or suspected of malnutrition and needed intervention for symptom management. A majority (82%) were overweight or obese according to BMI, indicating a higher risk of cancer recurrence. Participants’ dietary intake mainly consisted of carbohydrate- and protein-rich foods during treatment.

Conclusion: The side effects of chemotherapy varied among patients. The impact on body weight also differed, with some experiencing weight loss, others weight gain, and some no change. Regarding nutritional status, most patients were moderately malnourished, while a few were severely malnourished.

## Introduction

Cancer is a group of diseases caused by the development of abnormal cancerous cells that rapidly divide and form tumors. Tumors can either remain localized or spread to affect different parts of the body, referred to as benign or malignant tumors, respectively [1]. Cancer has become the second leading cause of deaths worldwide, with nearly 10 million deaths reported in 2020. The most common cancers are breast, colon, lung, rectum, and prostate cancers. Many cancers can be cured if detected early and treated effectively [2]. Cancers are categorized into stages to determine the tumor's location and progression in the body. Staging is typically done through physical examination, CT scans, and biopsy. Cancers are staged from 0 to 4, where stages 0 to 2 are usually referred to as early stages, in which tumors are localized and have not spread to other organs. Stages 3 and 4 are considered late stages because the cancer has spread to different parts of the body [3].

Breast cancer is a malignancy of breast tissue and is the second leading cause of deaths among women. Two genes commonly associated with breast cancer are BRCA1, BRCA2 (breast cancer-associated genes), and the HER2 gene (human epidermal growth receptor 2). Mutations and abnormal activity of these oncogenes can lead to the propagation of breast cancer [4]. Other risk factors include age, family history, giving birth to the first child after age 30, early menarche, genetic mutations, alcohol consumption, physical inactivity, obesity, diets high in fat, low intake of polyunsaturated fatty acids, low socioeconomic status, lack of awareness, elevated levels of certain hormones, and changes in dietary patterns [5]. According to the World Health Organization, 2.3 million new cases of breast cancer were diagnosed in 2020 and the numbers are increasing every year. Globally it accounts for 24.5% of all female cancer cases [6]. Compared to worldwide statistics, 45.4% of breast cancer cases were diagnosed in Asia [7]. In contrast to Western women, breast cancer in Asian women is often diagnosed at a younger age, typically between 40 and 50 years [7]. In Pakistan, breast cancer is the most prevalent cancer, accounting for 14.5% of all cancer cases in the country. One in eight Pakistani women is at risk of developing breast cancer at some stage of their life, with a mortality rate of 30.8% [8]. Data from the Punjab Cancer Registry report of 2022 indicate that 58.08% of cancer diagnoses among females in the region were breast cancer cases, which accounted for 44.7% of all cancers reported in women [8]. In 2021, breast cancer cases accounted for 47.3% of female cancers, highlighting an ongoing increase in the incidence of breast cancer [8].

Cancers are treated using either local therapies (such as radiotherapy, surgical removal of tumors, or immunotherapy targeting specific areas) or systemic therapies (such as chemotherapy, hormonal therapy, or immunotherapy for treating metastatic cancers). For triple-negative breast cancer, chemotherapy remains the primary systemic therapy, as hormonal therapy is effective for luminal A and B subtypes but not for triple-negative cases. This aligns with current evidence-based practices [9]. Chemotherapy for breast cancer can be recommended at any stage. For early-stage breast cancer, chemotherapy may be administered either before (neo-adjuvant) or after (adjuvant) surgery of the breast tumor. For metastatic breast cancer, chemotherapy aims to improve quality of life and slow tumor growth [10]. Chemotherapy drugs include alkylating agents (triazenes, nitrogen mustards, nitrosoureas, and platinum compounds), antitumor antibiotics (anthracyclines and non-anthracyclines), antimetabolites (enzyme inhibitors, antifolates, purine antagonists, and pyrimidine antagonists), mitotic inhibitors (taxanes and vinca alkaloids), and topoisomerase inhibitors [11]. While chemotherapy is effective in targeting cancerous cells, it also affects healthy cells, leading to side effects. Damage to healthy cells in areas such as the mouth and digestive tract often causes changes in dietary patterns. The side effects may include weight loss, fatigue, hair loss, mouth sores, loss of appetite, nausea, vomiting, pain while swallowing, throat problems, diarrhea, infection, and anemia, all of which can deteriorate a patient’s health status [12]. Some women with hormone receptor-positive breast cancer, particularly those undergoing hormonal therapy (e.g., tamoxifen or aromatase inhibitors), may experience weight gain during treatment. This weight gain has been identified as a potential risk factor for the recurrence of breast cancer, particularly in patients with a history of estrogen-sensitive tumors [13]. There is a lack of studies on the nutritional status of breast cancer patients in Pakistan. 

This study aims to evaluate the changes in dietary patterns of breast cancer patients during and after chemotherapy and to assess how the side effects of chemotherapy affect their nutritional status.

## Materials and methods

Study population

This cross-sectional study was conducted from September 2023 to December 2023. Breast cancer patients who had received a minimum of two sessions of chemotherapy were included in this study. Breast cancer patients from hospitals in Lahore participated voluntarily. Consent was obtained from patients who agreed to take part in this research. Data were collected from one public hospital (Jinnah Hospital). The study was conducted among 200 patients, and data were collected using a questionnaire and the Patient-Generated Subjective Global Assessment (PG-SGA) form.

Ethical statement

This study was approved by the Institutional Review Committee for Biomedical Research of the University of Veterinary and Health Sciences, Lahore, Pakistan. The research was conducted in compliance with the guidelines set by the committee. Prior to the commencement of the study, all participants provided informed consent to participate.

Inclusion criteria

Patients with breast cancer undergoing chemotherapy treatment were included in the study.

Exclusion criteria

Patients with cancers other than breast cancer and those receiving treatments other than chemotherapy were not included.

Data collection procedure

Data collection was performed using the PG-SGA form and a questionnaire (Appendix), which included additional questions. The first part of the form consisted of four sections focusing on weight history (changes in weight during the past two weeks or over the last one to six months), food intake (whether patients consumed more or less food than usual and the type of diet during treatment such as normal diet, liquid diet, or tube feeding), symptoms (e.g., loss of appetite, mouth sores, fatigue, loss of taste and smell, and gastrointestinal problems), and activities and function (activity levels after treatment). Scores for each section were calculated to assess the patient’s health. The second part of the form included details of diagnosis (cancer type and other co-morbidities), physical examination (muscle and fat loss, edema), and metabolic stress (fever intensity after treatment). Scores from both parts were summed to determine the nutritional status of the patients, which was categorized as well-nourished, moderately/suspected malnourished, or severely malnourished.

The questionnaire gathered sociodemographic information, details about the disease and treatment, and dietary recall. It included both open- and close-ended questions.

Sociodemographic information

Sociodemographic information was collected through a questionnaire. It included details about age, education, marital status, socioeconomic status, family type, and questions related to the disease and its treatment. Information was gathered on the type of cancer, breast cancer subtype and stage, family history, presence of risk factors, type of chemotherapy, number of sessions received and recommended, effect of chemotherapy on body weight, and use of any nutritional supplements. Participants were also asked about their diet type during the treatment period (normal, soft, or liquid diet) and the foods they frequently consumed to evaluate their dietary patterns.

PG-SGA scoring tool

The PG-SGA form, internationally recognized for screening the risk of malnutrition among cancer patients, was used for this study. It assessed patients’ weight history (loss or gain) over the past few weeks, food intake, symptoms following chemotherapy, physical activity, muscle or fat loss, fever, and any other coexisting conditions. Each section was scored individually, and the total score was calculated to evaluate the risk of malnutrition among the participants.

Statistical analysis

Data analysis was performed using IBM SPSS Statistics for Windows, Version 20 (Released 2011; IBM Corp., Armonk, New York). Descriptive statistics were applied to describe the demographic and clinical characteristics of the participants in terms of frequencies and percentages.

## Results

Sociodemographic variables

This study included 200 breast cancer patients, with an age range of 18 to 70 years and a mean age of 48 years. The majority of participants were female (99.4%) and married (73.5%). Most participants were housewives (85%), and a significant proportion were uneducated (67.5%). Regarding their living conditions, the majority resided in urban areas (76%) and lived in joint family systems (54.4%). The socioeconomic status of the majority of participants was categorized as low (60.5%). Table 1 presents self-reported sociodemographic data, including variables such as age, gender, marital status, education level, occupation, demographic area, family type, and socioeconomic status of breast cancer patients undergoing chemotherapy in Lahore. Values are expressed as numbers (percentages) unless stated otherwise. 

**Table 1:** Sociodemographic characteristics of breast cancer patients

Variables	Values
Mean age	48
Sociodemographic characteristics	n (%)
Gender	
Male	1(0.6%)
Female	199(99.4%)
Occupation	
Unemployed	8(4%)
Housewife	170(85%)
Working	20(10%)
Student	2(1%)
Marital status	
Married	147(73.5%)
Unmarried	6(3%)
Divorced	8(4%)
Widow	39(19.5%)
Educational level	
Uneducated	135(67.5%)
High school	49(24.5%)
Graduated	16(8%)
Demographic area	
Urban	152(76%)
Rural	48(24%)
Family type	
Joint	109(54.4%)
Nuclear	90(45%)
Socioeconomic status	
Low	121(60.5%)
Middle	79(39.5)
High	0(0%)

Types and determinants of breast cancer

The results of this study indicated that the majority of participants had localized breast cancer (57.5%). Invasive ductal carcinoma was the most prevalent type, observed in 45% of patients. Most participants (40%) were diagnosed with stage III breast cancer. Regarding potential risk factors, a positive family history of breast cancer was reported by 35% of participants. Additionally, 8.5% of participants reported the use of oral contraceptives, and 21% had not breastfed. Other factors such as pregnancy after the age of 30, no pregnancies, early menarche, and menopause were also identified as potential contributors to breast cancer development. The mean age of menarche among participants was 13 years. Postmenopausal breast cancer was reported in 56.5% of cases. Among 200 participants, 11 women had their first pregnancy between the ages of 31 and 40, while 9% reported no pregnancies. Table 2 provides detailed data on the type of cancer, stage of cancer, type of breast cancer, and its determinants.

**Table 2:** Type and determinants of breast cancer TNBC: triple-negative breast cancer, HER2+ve: human epidermal growth factor receptor 2 positive, HER2-ve: human epidermal growth factor receptor 2 negative, IDC: invasive ductal carcinoma

Variables	n (%)
Types of cancer	
Benign	115 (57.5%)
Metastatic	85 (42.5%)
Stages of cancer	
Stage 1	9 (4.5%)
Stage 2	59 (29.5%)
Stage 3	80 (40%)
Stage 4	52 (26%)
Types of breast cancer	
Triple-negative breast cancer (TNBC)	37 (18.5%)
HER2+ve	31 (15.5%)
HER2-ve	17 (8.5%)
Invasive ductal carcinoma (IDC)	90 (45%)
Invasive ductal carcinoma (triple positive)	18 (9%)
Invasive lobular carcinoma	2 (1%)
Invasive breast cancer	5 (2%)
Family history	
Yes	70 (35%)
No	130 (65%)
Use of oral contraceptives	
Yes	17 (8.5%)
No	166 (83%)
No pregnancy	17 (8.5%)
Breastfeeding	
Yes	141 (70.5%)
No	42 (21%)
No pregnancy	17 (8.5%)
Age of first pregnancy	
Below 20	53 (26.5%)
20-30	118 (59%)
31-40	11 (5.5%)
No pregnancy	18 (9%)
Mean age of menarche	13
Menopause	
Due to chemotherapy	46 (23%)
Not yet	41 (20.5%)
Post-menopausal	113 (56.5%)

Treatment-related variables

Table 3 contains data on the type of chemotherapy, route of administration, its effect on body weight, and the use of supplements or oral secretion agents for thinning saliva. The results showed that the majority of patients (60.5%) were given neoadjuvant chemotherapy. The route of administration was intravenous (100%). The mean number of chemotherapy sessions recommended and received among patients was 12 and 9, respectively. Out of 200 patients, only 61 (30.5%) reported using mineral and vitamin supplements during their treatment, with calcium supplements being the most common. Regarding changes in body weight, 45% of patients experienced weight loss, 24% had weight gain, and 31% reported no change in weight during treatment. Additionally, only 1.5% of patients used oral secretion agents for saliva thinning.

**Table 3:** Treatment-related variables

Variables	n (%)
Types of chemotherapy	
Neoadjuvant	121 (60.5%)
Adjuvant	79 (39.5%)
Route of chemotherapy	
IV route	160 (100%)
Mean sessions recommended	12
Mean sessions received	9
Use of supplements	
Yes	61 (30.5%)
No	136 (68%)
Weight loss during chemotherapy	
Yes	90 (45%)
No	48 (24%)
Unchanged	62 (31%)
Weight gain during chemotherapy	
Yes	48 (24%)
No	90 (45%)
Unchanged	62 (31%)
Use of oral secretion agent	
Yes	3 (1.5%)
No	197 (98.5%)

Side effects due to chemotherapy

Chemotherapy treatment had various side effects that impacted the nutritional status of breast cancer patients. These side effects included gastrointestinal problems, loss of appetite, changes in taste, mouth sores, dry mouth, fever, and fatigue. The results indicated that 10% of patients experienced difficulties while eating. Gastrointestinal symptoms such as vomiting (35%), nausea (30.5%), diarrhea (27.5%), and constipation (22.5%) were commonly reported during treatment. Other noted symptoms included loss of appetite (25%), anosmia (8.5%), ageusia (51%), fatigue (44%), mouth sores (20.6%), and dry mouth (23.8%). Additionally, 44% of patients experienced low-grade fever as a result of chemotherapy. Anemia was prevalent among participants, with 100 patients (50%) classified as mildly anemic, 35 patients (17.5%) as moderately anemic, and 2 patients (1%) as severely anemic. Other side effects included dizziness, peripheral numbness, swelling under the eyes, heartburn, dental problems, skin allergies, sweating, dryness, blurred vision, and edema. Patients also reported headaches, body aches, and bone weakness. Table 4 provides detailed data on the side effects of chemotherapy and their implications for nutritional status.

**Table 4:** Side effects of chemotherapy in breast cancer patients

Side effects	n (%)
Problem eating	
No	180 (90%)
Yes	20 (10%)
Loss of appetite	
No	150 (75%)
Yes	50 (25%)
Nausea	
No	131 (69.5%)
Yes	61 (30.5%)
Vomiting	
No	130 (65%)
Yes	70 (35%)
Diarrhea	
No	145 (72.5%)
Yes	55 (27.5%)
Constipation	
No	155 (77.5%)
Yes	45 (22.5%)
Mouth sores	
No	157 (78.5%)
Yes	42 (21%)
Dry mouth	
No	159 (79.5%)
Yes	41 (20.5%)
Anosmia (loss of smell)	
No	183 (91.5%)
Yes	17 (8.5%)
Ageusia (loss of taste)	
No	98 (49%)
Yes	102 (51%)
Fatigue	
No	112 (56%)
Yes	88 (44%)
Fever	
No	112 (56%)
Yes	88 (44%)
HB level	
Normal	63 (31.5%)
Mild anemia	100 (50%)
Moderate anemia	35 (17.5%)
Severe anemia	2 (1%)

Body mass index (BMI)

The body weight and height of the patients were measured, and the BMI was calculated. Results showed that 39.5% of participants were overweight and 2.5% of patients were underweight. The range of obesity class 1, class 2, and class 3 was noticed among 22.5%, 4.5%, and 2.5% of patients, respectively. Of the total participants, only 57 (28.5%) patients had normal BMI. Table 5 shows the classification of BMI. 

**Table 5:** Classification of body mass index

Body mass index (BMI)	Ranges (kg/m^2^)	n (%)
Underweight	Below 19	5 (2.5%)
Normal weight	19-24.9	57 (28.5%)
Overweight	25-29.9	79 (39.5%)
Obesity class 1	30-34.9	45 (22.5%)
Obesity class 2	35-39.9	9 (4.5%)
Obesity class 3	Above 40	5 (2.5%)

Nutritional triage recommendations

An additive scoring system was used to determine specific nutritional interventions for participants. These interventions included patient and family education, symptom management with pharmacological support, and appropriate nutritional strategies such as food-based interventions, nutritional supplements, enteral nutrition, or parenteral nutrition. The study results indicated that 60% of participants required immediate intervention to address and improve their symptoms. Table 6 outlines the different levels of interventions needed for symptom management among the participants.

**Table 6:** Nutritional triage recommendations

Levels of intervention	Range	n (%)
No intervention required	0-1	1 (0.5%)
Patient education by dietitian and pharmacological intervention is required.	2-3	8 (4%)
Requires intervention	4-8	71 (35.5%)
Indicates the critical need for improved symptom management	More than 9	120 (60%)

PG-SGA global assessment categories

According to the numerical score of this assessment form, the current nutritional status of patients is assessed and classified into three categories: Stage A (well-nourished), Stage B (moderate/suspected malnutrition), and Stage C (severely malnourished). This tool was used in this study, and the results showed that 36% of patients were well-nourished, 19.5% were suspected malnourished, and 29% were moderately malnourished. Out of 160 patients, only 31 (15.5%) were severely malnourished and required immediate nutritional treatment. Table 7 shows the nutritional status explained by different stages.

**Table 7:** Stages of nutritional status

Stages of nutritional status	n (%)
Stage A	
Well-nourished	72 (36%)
Stage B	
Suspected malnourished	39(19.5%)
Moderate malnourished	58 (29%)
Stage C	
Severely malnourished	31(15.5%)

Dietary choices during treatment

During treatment, the patients' diet was influenced by the side effects of chemotherapy. A dietary recall was conducted to assess their dietary patterns and choices during this period. The results indicated that the majority of participants opted for carbohydrate- and protein-rich foods. A preference for low-salt and non-spicy foods was also observed. Additionally, some participants reported cravings for crispy and fried foods. Table 8 presents the food items most commonly selected by the participants.

**Table 8:** Dietary choices during treatment

Dietary choices	n (%)
Chapati/bread	148 (74%)
Fruit/fruit juices	103 (51.5%)
Mutton/chicken/fish	81 (40.5%)
Pulses	121 (60.5%)
Vegetables	95 (45.5%)
Porridge	37 (18.5%)
Broth	58 (29%)
Milk/yogurt	120 (60%)
Egg	63 (31.5%)
Rice	103 (53.5%)
Nuts	20 (10%)

## Discussion

This is the first study of its kind assessing the impact of chemotherapy treatment on the nutritional status of breast cancer patients in Lahore. The results of this study showed that the majority of breast cancer patients belonged to urban areas, lived in joint families, and were from low socioeconomic status. The type of cancer in the majority of participants was localized. Most patients had invasive ductal carcinoma and were in the third stage of breast cancer. Risk factors reported by patients included a positive family history of breast cancer and lifestyle factors such as diet. The use of oral contraceptives and the practice of breastfeeding were noted among 8.5% and 21% of participants, respectively. In terms of treatment, neoadjuvant chemotherapy was the most commonly recommended type. The route of chemotherapy was intravenous (100%). Among 200 patients, 61 (30.5%) used mineral and vitamin supplements during their treatment, with calcium supplements being the most common. Weight loss was observed in 45% of patients, while 24% gained weight, and 31% experienced no weight change. Side effects reported included eating difficulties, vomiting, nausea, diarrhea, constipation, loss of appetite, anosmia, ageusia, fatigue, mouth sores, dry mouth, and low-grade fever. Half of the participants had mild anemia. Other symptoms noted included sweating, dizziness, numbness in hands and feet, headaches, body aches, and bone weakness. The BMI data showed that most participants were overweight, increasing the risk of cancer recurrence. The PG-SGA scoring tool revealed that most patients were moderately or suspected malnourished (Stage B). Among the participants, 60% scored above nine, indicating a need for immediate intervention to improve symptoms. Dietary recall showed that participants preferred carbohydrate- and protein-rich foods during treatment.

Neo-adjuvant chemotherapy is recommended on the basis of tumor size involvement of nodes and status of receptors. Patients who undergo neoadjuvant chemotherapy have increased chances of breast preservation and a decrease in the size of the tumor [14]. Chemotherapy affects the oral health of a patient. A study was conducted to determine the incidence of oral mucositis and risk factors in patients receiving outpatient chemotherapy. Data was collected through the “Mucositis grading scale” from 147 patients. Results showed that 51% of people were affected with mucositis, and 55.1% had mouth dryness and caused taste alterations [15]. A study from Brazil showed that anti-neoplastic treatments such as chemotherapy affect nutritional status. Data were collected four times during the study. Results of this study showed side effects of chemotherapy including reduction in food intake resulting in anorexia, nausea, vomiting, change in taste and smell, etc. Intake of carbohydrates and fat-containing foods were also reduced [16].

A cross-sectional study conducted in Ethiopia collected data from 281 patients receiving chemotherapy using the PG-SGA tool. The results showed that 58.2% of patients had malnutrition, 41.9% had moderate weight loss, 21.1% had severe weight loss, and 52.3% experienced weight loss in the past two weeks. Other side effects, such as loss of appetite, diarrhea, and nausea, were also observed [17]. The results of our study align with this study, as more than 60% of the patients in our study were malnourished. Women of young age and with an ideal weight are more susceptible to gaining weight during chemotherapy. According to the results of our study, 47% of women gained weight during treatment, with weight gain ranging from 1 to 6 kg. Treatment with anthracyclines without taxanes has been associated with an increased likelihood of weight gain [18]. Similarly, our study found that almost half of the patients were overweight.

A study was conducted to determine the effect of chemotherapeutic drugs on bone and muscle mass. It found that drugs like carboplatin directly affect muscle and bone mass, leading to muscle loss, while cisplatin leads to muscle wasting, with cancer cachexia being reported [19]. Another study examined the prevalence of anemia in breast cancer patients undergoing chemotherapy. Data from 292 patients revealed that 56% had mild anemia, 34% had moderate anemia, and 9% had severe anemia [20]. These results align with our findings, where more than 70% of the patients experienced anemia.

An interventional trial among breast cancer patients showed that food preferences change during and after chemotherapy. Women preferred high-protein foods and dairy products due to food aversions and altered chemosensory perception [21]. The results are consistent with our findings, where breast cancer patients showed a high intake of carbohydrates, such as bread, chapati, and rice, as well as dairy products like milk and yogurt, and protein-rich foods like eggs and meat. Another study analyzed the food preferences of breast cancer patients during treatment, examining taste detection thresholds (DTs) and recognition thresholds (RTs) for sweet, salty, bitter, and sour solutions. It reported a decline in sweet thresholds, while the thresholds for salty, bitter, and sour remained unchanged, with patients preferring mild and soft dishes [22]. Additionally, low dietary intake, including reduced consumption of fat and protein, was reported during adjuvant chemotherapy for early breast cancer [23]. Physical activity and exercise have been shown to improve fatigue and strength during treatment, while a neutropenic diet improves health outcomes but does not reduce infections or the adverse effects of chemotherapy [24].

Limitations

This cross-sectional study provides a snapshot of the side effects of chemotherapy, dietary patterns, its impact on nutritional status, and the prevalence of malnutrition among breast cancer patients. However, it does not address the long-term effects of chemotherapy concerning changes in dietary patterns and malnutrition. Additionally, the sample size in our study is not large enough to represent all breast cancer patients.

## Conclusions

This study concludes that the side effects of chemotherapy varied among breast cancer patients. The effect of chemotherapy on body weight also differed, with some patients experiencing weight loss, some gaining weight, and others showing no change. Regarding nutritional status, the majority of patients were found to be moderately malnourished.

This study highlights the importance of assessing the risk of malnutrition in breast cancer patients undergoing chemotherapy and underscores the need to develop nutritional interventions to mitigate the negative effects of treatment and improve patients’ quality of life. We recommend future longitudinal and interventional studies to generate robust evidence for targeted interventions.

## Appendices

 The questionnaire containing general information about the participants is presented in Table 9.

**Table 9:** General information

Name:	Contact no.:
Occupation:	Email ID:
Marital status	● Unmarried ● Married ● Widow
Age	● 18–25 ● 25–35 ● 35–50 ● Above 50
Level of education	● High school ● Undergraduate ● Graduate ● Other
Demographic area	● Urban ● Rural
Family type	● Joint family ● Nuclear family
Socioeconomic status	● Low class ● Middle class ● Upper class

Questions related to the disease and its treatment are given in Table 10.

**Table 10:** Disease- and treatment-related questions

Disease-related questions	
Type of cancer	● Benign ● Metastatic
Type of breast cancer	● Invasive ductal carcinoma ● Invasive lobular carcinoma ● Inflammatory breast cancer ● Triple-negative breast cancer
Family history	● Yes ● No
Did you use oral contraceptives?	● Yes ● No
Did you breastfeed your kids?	● Yes ● No Other
When did you have your first pregnancy?	● Below 30 ● Between 30-35 ● Above 35
Age of menarche	
At what age, did you have your menopause	
Treatment-related questions	
Type of chemotherapy	
Routes of chemotherapy	
Sessions of chemotherapy recommended?	
Sessions of chemotherapy received to date	
Do you experience weight loss during the treatment	● Yes ● No
Do you experience weight gain during the treatment	● Yes ● No
How well are you able to deal with your stress?	● Yes ● No
Do you get any encouragement from your family or friends?	● Yes ● No
Do you use any vitamin, mineral, or fiber supplements?	● Yes ● No; If yes, please specify
Do you take any oral secretion agent for the thinning of saliva?	● Yes ● No

The 24-hour dietary recall questionnaire used in this study is given in Table 11.

**Table 11:** Twenty-four-hour dietary recall Caloric requirements were calculated using the Harris-Benedict equation. Protein requirement was calculated by using reference values given in Krause (food and nutrition process).

Time of meal	Food item	Quantity	Method of preparation
Breakfast			
Snack			
Lunch			
Snack			
Dinner			

Current intake	Required intake
Calories =	Calories =
Protein =	Protein =
Fat =	Fat =
Carbohydrates =	Carbohydrates =
Fluid intake =	Fluid intake =
